# Sparse CT reconstruction based on multi-direction anisotropic total variation (MDATV)

**DOI:** 10.1186/1475-925X-13-92

**Published:** 2014-07-04

**Authors:** Hongxiao Li, Xiaodong Chen, Yi Wang, Zhongxing Zhou, Qingzhen Zhu, Daoyin Yu

**Affiliations:** 1 College of Precision Instrument and Opto-Electronics Engineering, Tianjin University, Tianjin 300072, People’s Republic of China; 2 Key Laboratory of Opto-Electronics Information Technology of Ministry of Education, Tianjin University, Tianjin 300072, People’s Republic of China; 3 Tianjin Key Laboratory of Biomedical Detecting Techniques and Instruments, Tianjin University, Tianjin 300072, People’s Republic of China

**Keywords:** Sparse CT, Iterative reconstruction, Anisotropic total variation, Coordinate rotation transform, Edges in multiple directions

## Abstract

**Background:**

The sparse CT (Computed Tomography), inspired by compressed sensing, means to introduce a prior information of image sparsity into CT reconstruction to reduce the input projections so as to reduce the potential threat of incremental X-ray dose to patients’ health. Recently, many remarkable works were concentrated on the sparse CT reconstruction from sparse (limited-angle or few-view style) projections. In this paper we would like to incorporate more prior information into the sparse CT reconstruction for improvement of performance. It is known decades ago that the given projection directions can provide information about the directions of edges in the restored CT image. ATV (Anisotropic Total Variation), a TV (Total Variation) norm based regularization, could use the prior information of image sparsity and edge direction simultaneously. But ATV can only represent the edge information in few directions and lose much prior information of image edges in other directions.

**Methods:**

To sufficiently use the prior information of edge directions, a novel MDATV (Multi-Direction Anisotropic Total Variation) is proposed. In this paper we introduce the 2D-IGS (Two Dimensional Image Gradient Space), and combined the coordinate rotation transform with 2D-IGS to represent edge information in multiple directions. Then by incorporating this multi-direction representation into ATV norm we get the MDATV regularization. To solve the optimization problem based on the MDATV regularization, a novel ART (algebraic reconstruction technique) + MDATV scheme is outlined. And NESTA (NESTerov’s Algorithm) is proposed to replace GD (Gradient Descent) for minimizing the TV-based regularization.

**Results:**

The numerical and real data experiments demonstrate that MDATV based iterative reconstruction improved the quality of restored image. NESTA is more suitable than GD for minimization of TV-based regularization.

**Conclusions:**

MDATV regularization can sufficiently use the prior information of image sparsity and edge information simultaneously. By incorporating more prior information, MDATV based approach could reconstruct the image more exactly.

## Background

CT (Computed Tomography) is one of the most important medical image technologies. Due to the potential cancer risk associated with the radiation dose in CT, recent technical development focuses on reducing radiation dose in CT. Several CT machine manufacturers have developed CT machines with iterative reconstruction algorithms to reduce radiation dose, such as the IRIS (Iterative Reconstruction in Image Space) of Siemens, ASiR (Adaptive Statistical Iterative Reconstruction) of GE (General Electric Co.), iDose (a trademark) of Philips and AIDR (Adaptive Iterative Dose Reduction) 3-D of Toshiba, which are reported to reduce the radiation dose by 30-50% while maintaining the reconstructed image quality comparable to the conventional FBP (Filtered Back Projection) method [1-5].

On the other hand, biomedical engineering researchers are trying to improve the performance of the iterative reconstruction algorithms by introducing more prior information of the reconstructed images to reduce the projection input and the radiation dose even more. One important prior information is image sparsity, whose usefulness has been testified by the recent popular compressed sensing technique in signal processing [6-8]. Before the compressed sensing, the sparsity of medical images has been used for limited-angle tomography [9] and few-view CT blood-vessel reconstruction [10]. Then following the compressed sensing theory, two kinds of sparse models were proposed for the sparse CT reconstruction. By ‘sparse CT’ we mean two aspects. First, a single CT image is approximately sparse like other natural images, and the information changes between successive CT images in dynamic CT are also approximately sparse. Second, the projection data is sparsely collected, such as limited-angle tomography [9] and few-view CT [10], rather than the full view dense collection in conventional CT scan. The first model uses the sparsity coming from the gradient transformation of the image which is assumed to be approximately piecewise-constant, such as ART-TV-MIN (Algebraic Reconstruction Technique Total Variation MINimization) [11] and ASD-POCS (Adaptive Steepest Descent Projection Onto Convex Sets) [12]. The second model uses the sparsity coming from the subtraction of the reconstructed image from its prior image, such as PICCS (Prior Image Constrained Compressed Sensing) [13], PIRPLE (Prior Image Registered Penalized Likelihood Estimation) [14,15] and FCCS (Feature Constrained Compressed Sensing) [16].

In this paper we consider the methods based on the first model for the sparse CT reconstruction problems. In this model, the reconstruction problem can be written as a constrained optimization

(1) $$\text{min}{||\vec{f}||}_{\text{reg}}\;\text{subject}\;\mathrm{to}\;ℳ\vec{f}\;=\;\vec{g}$$

The equality constraint is a guarantee of the data fidelity, where ℳ is the projection matrix modeling the forward projection, $\vec{f}$ is the image vector to be restored, and $\vec{g}$ is the projection vector. The objective function is a norm function of $\vec{f}$, which is a regularization for introducing some prior information like image sparsity. By using norm function of $\vec{f}$, we want to obtain a sparse solution of this constrained optimization. Therefore, sparser $\vec{f}$ is, smaller ${||\vec{f}||}_{\text{reg}}$ should be. A commonly used norm function is TV (Total Variation) norm [11]. The optimization problem can also be written as an unconstrained form

(2) $$\text{arg}\;\text{min}\;\left[{\left(ℳ\vec{f}-\vec{g}\right)}^{2}+\lambda {{||\vec{f}||}^{2}}_{\text{reg}}\right]$$

where λ  is a regularization parameter adjusting the relative penalties on the sparseness and the data fidelity.

To promote the performance of sparse CT reconstruction, two kinds of improvements are often considered. The first kind of improvement focuses on the sparsity regularization. Sidky [17] proposed the sparser TpV (Total p-Variation) norm to replace the TV norm. Yu [18] used the Haar wavelet transformation to make use of sparsity. Jia [19] replaced the TV norm with tight frame and used GPU (Graphics Processing Unit) to accelerate the reconstruction speed. Apart from replacement of TV norm, Tian [20], Liu [21], and Chang [22] developed some adaptive adjustment factors embedded in the TV norm to enhance the edge characteristic. Recently, Liu [23] introduced the TV-Stokes regularizer into sparse CT, which can smooth the isophote in the target image so as to preserve the detail and smoothness of the reconstructed image. The second kind of improvement focuses on the algorithms for resolving (2), which include CG (Conjugate Gradient) [24], forward backward splitting plus GPU [25], split-bregman [26], GPBB (Gradient Projection Barzilai Borwein) plus GPU [27], Chambolee-Pock algorithm [28], and ADMM (Alternating Direction Method of Multipliers) [29]. Besides, Niu and Zhu [30] proposed to use the log-barrier term to approximate the data fidelity term, which removes the forward and backward projection in the traditional iterative algorithms like ART (Algebraic Reconstruction Technique).

In this paper, we consider the first kind of improvement. The MDATV (Multi-Direction Anisotropic Total Variation) is proposed to replace TV norm. The aim of MDATV is to introduce into sparse CT reconstruction more prior information of edges, as the ATV (Anisotropic Total Variation) does [31,32]. But ATV can only represent the edge information in few directions, which loses much prior information of edge directions. This paper combines the coordinate rotation transform with 2D-IGS (Two Dimensional Image Gradient Space), and represents the edge information in multiple directions easily. Based on this multi-direction representation, MDATV can incorporate more edge information into the sparse CT reconstruction, so as to improve the performance.

The rest of the paper is organized as follows. In section Methods, firstly, the background of ART and ATV is reviewed. Then the MDATV norm and its corresponding minimization methods are described. Finally, the ART + MDATV scheme is outlined. Section Simulations and experiments uses the proposed MDATV based approach to solve sparse CT reconstruction problems in numerical simulations and with experimental data, so as to demonstrate its remarkable efficiency. Finally, we conclude with section Discussions and conclusions, discussing the pros and cons of MDATV.

## Methods

### Review of ART + ATV

#### ***ART***

The ART + ATV is proposed based on ART + TV method [11,12]. ART updates the estimated image iteratively. Firstly, the estimated image is forward projected into the sinogram space, then the difference between the estimated sinogram and the given projections is back projected into the image space to update the estimated image. These steps are operated iteratively until some termination criteria are met. This method is also known as POCS (Projection Onto Convex Sets) in linear algebra. The ART update formula is as following

$$\vec{f}\left[n,m+1\right]=\vec{f}\left[n,m\right]+{\vec{M}}_{m}^{T}\frac{g_{m}-{\vec{M}}_{m}\;.\;\vec{f}\left[n,m\right]}{{\vec{M}}_{m}\;.\;{\vec{M}}_{m}^{T}}$$

where *g*_*m*_ is the *m*th element of the projection $\vec{g}$. ${\vec{M}}_{m}$ is the *m*th row vector of the projection matrix ℳ. $\vec{f}\left[n,m\right]$ is the estimated image after fusing the *m*th element of the projection data during the *n*th iteration. The superscript *T* represents transpose.

#### ***TV and ATV***

The TV norm in the ART + TV method is used to introduce and strengthen the prior information of image sparsity. This prior information significantly improves the quality of reconstructed image with low computation load. The TV norm is defined as

(3) $${||\vec{f}||}_{\mathrm{TV}}\;:=\;\sum_{i,j}{||\nabla f_{i,j}||}_{\ell_{2}}=\sum_{i,j}\sqrt{{\left(D_{i}f_{i,j}\right)}^{2}\;+\;{\left(D_{i}f_{i,j}\right)}^{2}}$$

where *f*_*i,j*_ is the pixel on the *i*th row and the *j*th column in the target image. *D*_*i*_*f*_*i,j*_*=f*_*i,j*_*-f*_*i–1,j*_, and *D*_*j*_*f*_*i,j*_*=f*_*i,j*_*-f*_*i,j–1*_ are the vertical and the horizontal gradients respectively, *D*_*i*_ and *D*_*j*_ are the corresponding finite differential operators. Because the image edges are orthogonal to their corresponding gradients, *D*_*i*_*f*_*i,j*_ and *D*_*j*_*f*_*i,j*_ can also represent edges in the horizontal and the vertical directions respectively. The TV norm is isotropic, since it assigns the same energy to both the vertical and the horizontal gradients. Different from TV norm, ATV norm is anisotropic and defined as

(4) $${||\vec{f}||}_{\text{ATV}}\;:=\;\sum_{i,j}{||\nabla_{\text{A,B}}f_{i,j}||}_{\ell_{2}}\;=\sum_{i,j}\sqrt{A{\left(D_{i}f_{i,j}\right)}^{2}\;+\;B{\left(D_{i}f_{i,j}\right)}^{2}}$$

where *A* and *B* are weights controlling the energy ratios of the vertical and the horizontal gradients, respectively. When *A = B*, it is isotropic, else it is anisotropic. (4) can also be written as

$${||\vec{f}||}_{\text{ATV}}\;:=\sum_{i,j}{||\nabla_{\eta }f_{i,j}||}_{\ell_{2}}\;=\;\sum_{i,j}\sqrt{\eta {\left(D_{i}f_{i,j}\right)}^{2}\;+\;{\left(D_{i}f_{i,j}\right)}^{2}}$$

where *η=A/B* is the weight factor. Therefore, we only need one parameter to control the energy ratios of the vertical and the horizontal gradients without affecting the results of optimization. In this paper we set *η*=1000. Based on experiments, this value of *η* can make the edges in the enhanced direction strong enough, while the potential artifacts in the suppressed direction weak enough. When *η* is smaller, the suppression to the potential artifacts may be not sufficient. A bigger *η* is also valid, but the reconstructions hardly change any more.

#### ***Motivation of ATV***

The motivation of ATV is to introduce into sparse CT reconstruction a prior information of edge directions. This prior information was discovered by Quinto [33] decades ago. In sparse CT reconstruction, the edge information tangent to the projection rays would be measured and restored easily, while those not tangent to the projection rays should be harder to ‘see’. This phenomenon can be theoretically explained by the central slice theory [34].

**Figure 1:**
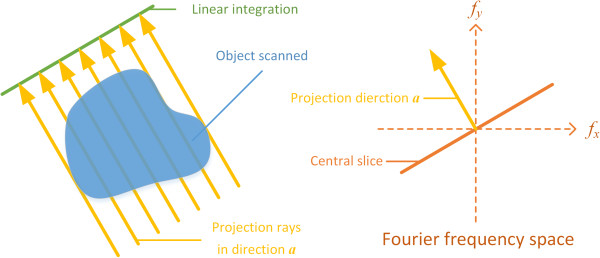
Central slice theory.

Without loss of generality, we take the parallel projection for example. This example is shown in Figure 1. Suppose the projection rays in direction ***a*** produce a row of projections. Each point of the projection row corresponds to one projection ray, and each point’s value is the linear integration of the attenuation coefficients passed through by the corresponding projection ray. According to the central slice theory, the 1-D Fourier transformation of this projection row is the central slice perpendicular to ***a*** in the Fourier frequency space. In the 2D Fourier space, the central slice is a line passing through the origin. Thereby the projection data collected by projection rays in direction ***a*** corresponds to a central slice perpendicular to ***a*** in the 2D Fourier space.

**Figure 2:**
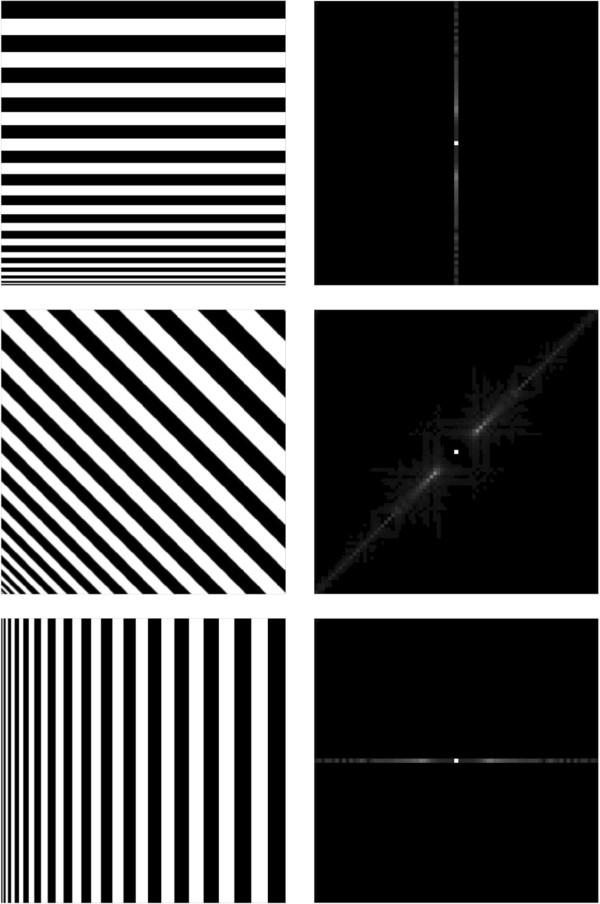
Edges’ direction in 2D image (left column) is perpendicular to their corresponding Fourier transformation’s direction (right column).

According to the Fourier optics [35], if a family of edges in a 2D image are in the direction ***a***, then the Fourier transformation of these edges is a line passing through the origin and perpendicular to ***a*** in the 2D Fourier frequency space, as shown in Figure 2. This means that the line passing through the origin and perpendicular to ***a*** in the 2D Fourier space corresponds to the edge information in direction ***a*** of the 2D image.

Compare Figures 1 and 2, we find that the projection rays in direction ***a*** record the edge information in the same direction. Therefore, the edge information tangent to the projection rays would be restored easily, since these edge information is well recorded by the projection rays. While the edge information perpendicular to the projection rays is not recorded, thereby these edges are hard to ‘see’.

The discussion above indicates that the geometrical information of projection may affect the sparse CT reconstruction. For example in the limited-angle projection, if most projections are tangent or approximately tangent to the horizontal direction, then the isotropic TV regularization may result in some artifacts in the vertical direction, which will blur the clear edges in the horizontal direction. Since the projections did not record any edge information in the vertical direction, the energy vacancy in the vertical direction would be filled by artifacts. However, ATV could handle this problem by assigning more energy in the horizontal direction and less energy in the vertical direction, thus to enhance the edge information in the horizontal direction and suppress the artifacts in the vertical direction.

### MDATV

However, ATV can only represent edges in few directions. This may lose much prior information of edges in many other directions. To remedy this defect, MDATV is proposed to use the entire prior information of edges.

#### ***2D-IGS***

**Figure 3:**
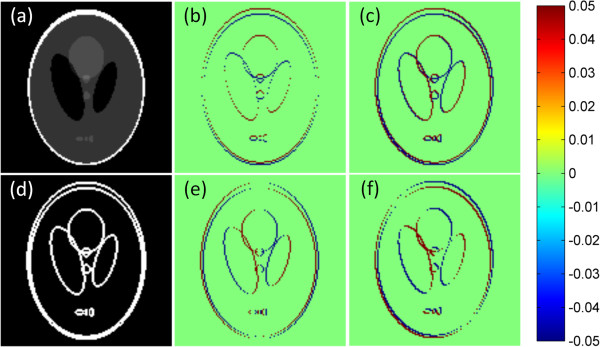
**The Shepp-Logan phantom and edges. (a)** The original Shepp-Logan phantom with display gray scale window of [0,1], (b) its horizontal edges *E*_*h*_, **(c)** rotated horizontal edges *E*_*hr*_, **(d)** gradient image with display gray scale window of [0,0.01], and **(e)** vertical edges *E*_*v*_, **(f)** rotated vertical edges *E*_*vr*_. The edges are rotated counterclockwise by 45°.

Before description of MDATV, we firstly introduce the 2D-IGS. In a 2D discrete image, *f*_*i,j*_ is defined as the pixel on the *i*th row and the *j*th column. The 2D-IGS Ψ  contains two parts. One is the vertical edges denoted by the 2D matrix ***E***_***v***_, and the other is the horizontal edges denoted by the 2D matrix ***E***_***h***_. They are defined as

(5) $$\left\{{}_{E_{v}\left[i,j\right]\;=\;D_{h}f_{i,j}\;=\;f_{i,j}\;-\;f_{i,j-1}}^{E_{h}\left[i,j\right]\;=\;D_{v}f_{i,j}\;=\;f_{i,j}\;-\;f_{i-1,j}}\right)$$

where *D*_*v*_ and *D*_*h*_ are the same as *D*_*i*_ and *D*_*j*_ in (3) representing the vertical and the horizontal finite differential operators respectively. The ***E***_***h***_ and ***E***_***v***_ of Shepp-Logan phantom are shown in Figure 3(b) and (e). The original image is generated by the function ‘phantom(128)’ in MATLAB^®^, as shown in Figure 3(a).

#### ***Edges in multiple directions***

***E***_***h***_ and ***E***_***v***_ represent the horizontal and the vertical edges of the 2D image, which are used in the TV and ATV norm. To represent edges in many more other directions, the coordinate rotation transform is introduced to be applied to the 2D-IGS.

**Figure 4:**
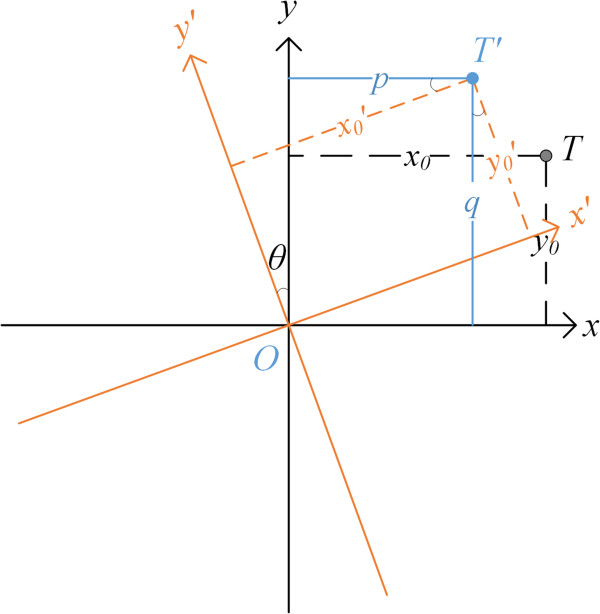
**Coordinates system *xOy *rotates counterclockwise by *θ *degrees to coordinate system *x***′***Oy′.***

First, we recall the elementary coordinate rotation transform in 2D space. Figure 4 shows the transform process. The coordinate system *xOy* rotates counterclockwise by *θ* degrees to the coordinate system *x*′*Oy*′, where 0^*°*^ ≤ *θ* ≤ 180^*°*^. The target point *T* in *xOy* is denoted by (*x*_0_, *y*_0_). Its corresponding rotated target point *T*′ is denoted by $\left(x_{0}^{\prime },y_{0}^{\prime }\right)$ in *x*′*Oy*′. Obviously, we have $x_{0}=x_{0}^{\prime }$ and $y_{0}=y_{0}^{\prime }$. When we need to process the rotated target point *T*′ in *xOy*, we can project the coordinates of *T*′ in *x*′*Oy*′ onto the coordinate system *xOy*. Let (*p*, *q*) denote the projected coordinates of *T*′ in *xOy*. According to the elementary geometry, the transform formula is

(6) $$\left\{\begin{array}{l} p=x_{0}^{\prime }\cdot \text{cos}\theta -y_{0}^{\prime }\cdot \text{sin}\theta =x_{0}\cdot \text{cos}\theta -y_{0}\cdot \text{sin}\theta \\ q=x_{0}^{\prime }\cdot \text{sin}\theta +y_{0}^{\prime }\cdot \text{cos}\theta =x_{0}\cdot \text{sin}\theta +y_{0}\cdot \text{cos}\theta \end{array}\right)$$

where *p* is the linear combination of the projections of $x_{0}^{\prime }$ and $y_{0}^{\prime }$ onto the *x* axis. *q* is the linear combination of the projections of $x_{0}^{\prime }$ and $y_{0}^{\prime }$ onto the *y* axis.

When the coordinate rotation transform is applied to the 2D-IGS Ψ , the horizontal edges ***E***_***h***_ corresponds to the horizontal line segment *x*_0_ in (6), and the vertical edges ***E***_***v***_ corresponds to the vertical line segment *y*_0_ in (6). After counterclockwise rotation by θ  degrees, we get the rotated 2D-IGS **Ψ**′, which contains the rotated horizontal and vertical edges $E_{h}^{\prime }$ and $E_{v}^{\prime }$. But we need to process the rotated horizontal and vertical edges in the 2D-IGS Ψ . Therefore, we need to project $E_{h}^{\prime }$ and $E_{v}^{\prime }$ onto the 2D-IGS Ψ . Let ***E***_***hr***_ and ***E***_***vr***_ denote the projected $E_{h}^{\prime }$ and $E_{v}^{\prime }$. Then ***E***_***hr***_ and ***E***_***vr***_ correspond to *p* and *q* in (6). Thus we have

(7) $$\left\{{}_{E_{\mathrm{vr}}\left[i,j\right]\;=\;E_{h}\left[i,j\right]\;\cdot \;\text{sin}\theta \;-\;E_{v}\left[i,j\right]\;\cdot \;\text{cos}\theta }^{E_{\mathrm{hr}}\left[i,j\right]\;=\;E_{h}\left[i,j\right]\;\cdot \;\text{cos}\theta \;-\;E_{v}\left[i,j\right]\;\cdot \;\text{sin}\theta }\right)$$

By adjusting the angle θ , the edges in any direction could be denoted by (7). The rotated edges ***E***_***hr***_ and ***E***_***vr***_ of Shepp-Logan phantom are shown in Figure 3(c) and (f) with the rotation angle of –45°. Based on (5), (7) could be rewritten by the finite differential operators

$$\left\{{}_{D_{\mathrm{hr}}\left[i,j\right]\;=\;D_{v}\left[i,j\right]\;\cdot \;\text{sin}\theta \;-\;D_{h}\left[i,j\right]\;\cdot \;\text{cos}\theta }^{D_{\mathrm{vr}}\left[i,j\right]\;=\;D_{v}\left[i,j\right]\;\cdot \;\text{cos}\theta \;-\;D_{h}\left[i,j\right]\;\cdot \;\text{sin}\theta }\right)$$

where ***D***_***vr***_ and ***D***_***hr***_ are the rotated vertical and horizontal finite differential operators.

#### ***MDATV***

Based on the multi-direction representation, the MDATV is defined as

(8) $${||\vec{f}||}_{\text{MDATV}}:=\;\sum_{i,j}\sqrt{\eta {\left(E_{h}\left[i,j\right]\;\text{cos}\theta \;-\;E_{v}\left[i,j\right]\;\text{sin}\theta \right)}^{2}\;+\;{\left(E_{h}\left[i,j\right]\;\text{sin}\theta \;-\;E_{v}\left[i,j\right]\;\text{cos}\theta \right)}^{2}\;}$$

where ***E***_***h***_ and ***E***_***v***_ are defined in (5), η  is the weights adjusting the the energy ratios of the rotated horizontal and vertical edges. Substitute (7) into (8), we get the simplified form of (8)

$${||\vec{f}||}_{\text{MDATV}}:=\;\sum_{i,j}\sqrt{\eta {\left(E_{\mathrm{hr}}\left[i,j\right]\right)}^{2}\;+\;{\left(E_{\mathrm{vr}}\left[i,j\right]\right)}^{2}\;}$$

In this paper we set η  = 1000 with the same reason in ATV. Therefore, the rotated horizontal edges ***E***_***hr***_ are representing the edges parallel to the projection rays. If η  < 1, then the rotated vertical edges ***E***_***vr***_ would represent the edges parallel to the projection rays.

In optimization problem (1), the objective function is some kind of norm function, such as TV, ATV or MDATV. Whatever the objective function is, the optimization requires to minimize this function. One common method for minimization is gradient descent (GD). Thus we need to compute the gradient of MDATV. Substitute (5) into (8) and the MDATV’s gradient is

(9) $$\begin{array}{l} \frac{\partial {||\vec{f}||}_{\text{MDATV}}}{\partial f_{i,j}}\;=\;\frac{\eta \cdot \left(\text{cos}\theta -\text{sin}\theta \right)\cdot \left(E_{h1}\text{cos}\theta -E_{v1}\text{sin}\theta \right)\;+\;\left(\text{sin}\theta +\text{cos}\theta \right)\cdot \left(E_{h1}\text{sin}\theta +E_{v1}\text{cos}\theta \right)}{\sqrt{\eta \cdot {\left(E_{h1}\text{cos}\theta -E_{v1}\text{sin}\theta \right)}^{2}\;+\;{\left(E_{h1}\text{sin}\theta +E_{v1}\text{cos}\theta \right)}^{2}\;+\;\in }}\;\\ \;+\;\frac{-\eta \cdot \text{cos}\theta \cdot \left(E_{h2}\text{cos}\theta -E_{v2}\text{sin}\theta \right)\;-\;\text{sin}\theta \cdot \left(E_{h2}\text{sin}\theta +E_{v2}\text{cos}\theta \right)}{\sqrt{\eta \cdot {\left(E_{h2}\text{cos}\theta -E_{v2}\text{sin}\theta \right)}^{2}\;+\;{\left(E_{h2}\text{sin}\theta +E_{v2}\text{cos}\theta \right)}^{2}\;+\;\in }}\;\\ \;+\;\frac{\eta \cdot \text{sin}\theta \cdot \left(E_{h3}\text{cos}\theta -E_{v3}\text{sin}\theta \right)\;-\;\text{cos}\theta \cdot \left(E_{h3}\text{sin}\theta +E_{v3}\text{cos}\theta \right)}{\sqrt{\eta \cdot {\left(E_{h3}\text{cos}\theta -E_{v3}\text{sin}\theta \right)}^{2}\;+\;{\left(E_{h3}\text{sin}\theta +E_{v3}\text{cos}\theta \right)}^{2}\;+\;\in }} \end{array}$$

where

$$\left\{\begin{array}{l} E_{h1}=f_{i,j}-f_{i,j-1}\\ E_{v1}=f_{i,j}-f_{i-1,j}\\ E_{h2}=f_{i,j+1}-f_{i,j}\\ E_{v2}=f_{i,j+1}-f_{i-1,j+1}\\ E_{h3}=f_{i+1,j}-f_{i+1,j-1}\\ E_{v3}=f_{i+1,j}-f_{i,j} \end{array}\right)$$

and *ϵ* is a small positive constant to avoid the singularity. In this paper we set *ϵ* = 1.0 × 10^- 8^.

### Minimization approaches

#### ***GD (Gradient Descent)***

The workflow of GD for minimizing regularization in optimization (1) is as following:

a) Compute the balancing parameter between ART (data fidelity) and GD (regularization)

$$d\;:=\;{||{\vec{f}}^{\left(J\right)}\;-\;{\vec{f}}^{\left(0\right)}||}_{2}$$

b) for *k* = 1,2,… *K*

$${\vec{f}}^{\left(J,0\right)}\;=\;{\vec{f}}^{\left(J\right)}$$

$$\vec{v}\;=\;\frac{\partial {||\vec{f}||}_{\text{reg}}}{\partial f_{\mathrm{ij}}}$$

$$\overset{\wedge }{v}\;=\;\frac{\vec{v}}{{\left|\vec{v}\right|}^{2}}$$

$${\vec{f}}^{\left(J,k\right)}\;=\;{\vec{f}}^{\left(J,k-1\right)}\;-\;a\cdot d\cdot \overset{\wedge }{v}$$

end for

Where ${\vec{f}}^{\left(0\right)}$ and ${\vec{f}}^{\left(J\right)}$ are the estimated image before and after the *J*th iteration of ART respectively, and α  is the step size. Although the variable *d* is kind of useful for adaptively adjusting the step size of GD, α  is determinant in practice.

Note that the norm of $\vec{v}/{\left|\vec{v}\right|}^{2}$ is smaller than the norm of $\vec{v}/\left|\vec{v}\right|$. In GD there usually is a fixed best step size α * such that the convergence rate is fastest. This α * may be found by the traversal method, which tries many step sizes for the problem and finds the best one within a given accuracy. Obviously, smaller the gradient vector $\overset{\wedge }{v}$ is, bigger the best step size α * is. The bigger step size is more resistant to the perturbation. For example, the perturbation of 0.1 is negligible for the step size of 50, but this perturbation would have a significant impact for the step size of 0.01. Therefore, we use $\vec{v}/{\left|\vec{v}\right|}^{2}$ instead of $\vec{v}/\left|\vec{v}\right|$ as the gradient vector $\overset{\wedge }{v}$ in this paper.

However, in optimization (1), GD is not a stable method for minimizing TV, ATV or MDATV regularizations. Since the best step size of GD changes greatly with the projection parameters and the target images. And the imperfect step size may greatly slow down the convergence rate and degenerate the quality of the estimated image. To overcome this defect of GD, the NESTA (NESTerov’s algorithm) [36] method is proposed to minimize the regularizations in (1).

#### ***NESTA***

Because the MDATV could be expressed as

(10) $$\begin{array}{l} {||\vec{x}||}_{\text{MDATV}}\;=\;\sum_{i,j}\sqrt{\eta {\left(E_{\mathrm{hr}}\left[i,j\right]\right)}^{2}\;+\;{\left(E_{\mathrm{vr}}\left[i,j\right]\right)}^{2}}\\ \;=\sum_{i,j}\sqrt{{\left(E{'}_{\mathrm{hr}}\left[i,j\right]\right)}^{2}\;+\;{\left(E{'}_{\mathrm{vr}}\left[i,j\right]\right)}^{2}}\\ \; \end{array}$$

where $E{'}_{\mathrm{hr}}\;=\sqrt{\eta }E_{\mathrm{hr}},\cdot E{'}_{\mathrm{vr}}\;=\;E_{\mathrm{vr}}$. The form of MDATV norm in (10) is the same as the TV norm in (3), which is almost the similar for ATV and TV. Thus we can first study the NESTA method for TV minimization and then apply the conclusions to ATV and MDATV minimization.

NESTA, based on the Nesterove’s smoothing technique [37], is a fast first-order method for sparse recovery. First, the nonsmooth TV norm can be rewritten as

(11) $${||\vec{f}||}_{\mathrm{TV}}\;=\;\sum_{i,j}\underset{u\in Qd}{\max\langle u,Df_{i,j}\rangle }$$

where $\vec{f}\in Q_{p}$, the convex set $Q_{p}$ is referred to as the primal feasible set. *u* = [*u*_1_, *u*_2_]^*T*^ is in the dual feasible set $Q_{d}$ if and only if $u_{1}^{2}\left[i,j\right]\;+\;u_{2}^{2}\left[i,j\right]\;\leq \;1$, and *Df*_*i,j*_ = [*D*_*h*_*f*_*i,j*_*,D*_*v*_*f*_*i,j*_]^*T*^ is the finite difference of the 2D image *f*_*i,j*_. The superscript *T* denotes transpose. If *u* is regarded as a 2D vector, then $Q_{d}$ is a *ℓ*_*2*_ norm unit ball.

According to Nesterov’s work, the smoothed TV regularization function is (12)

where μ  should be set sufficiently small such that . Here we set μ  = 0.01. Then  is given by(13)

Where *D* = [*D*_*h*_*,D*_*v*_]^*T*^, $u_{u}\left(\vec{f}\right)$ is of the form [*u*_*1*_*,u*_*2*_]^*T*^ and for each *a* ∈ {*h*, *v*},

$$u_{a}\left[i,j\right]\;=\;\left\{\begin{array}{c} \mu^{-1}\left(D_{a}\vec{f}\right)\left[i,j\right]\\ {||\nabla f\left[i,j\right]||}_{\ell_{2}}^{-1}\left(D_{a}\vec{f}\right)\left[i,j\right] \end{array}\;\begin{array}{c} \mathrm{if}{||\nabla f\left[i,j\right]||}_{\ell_{2}}\;<\;\mu \\ \mathrm{otherwise} \end{array}\right)$$

The minimization of the smoothed TV norm by Nesterov’s algorithm is obtained by iteratively estimating three sequences $\left\{{\vec{f}}_{k}\right\}$, $\left\{{\vec{d}}_{k}\right\}$, and $\left\{{\vec{e}}_{k}\right\}$ as follows:

Initialize $\vec{f_{0}}$. for *k = 1,2,…,K*,

a) Compute 

b) Compute ${\vec{d}}_{k}$

(14)

c) Compute ${\vec{e}}_{k}$

(15)

d) Update ${\vec{f}}_{k}$

(16) $${\vec{f}}_{k+1}\;=\;\tau_{k}{\vec{e}}_{k}\;+\;\left(1-\tau_{k}\right){\vec{d}}_{k}$$

end for.

In the above algorithm, *α*_*i*_ = (*i* + 1)/2, *τ*_*k*_ = 2/(*k* + 3) is suggested for fast convergence. Since the goal is minimizing TV norm without other constraints, ${\vec{d}}_{k}$ and ${\vec{e}}_{k}$ can be computed by letting the gradients of the object functions be zero and then we get 

(17)

In (15), a suitable smoothing prox-function is

$$p_{p}\left(\vec{f}\right)\;=\;\frac{1}{2}{||\vec{f}\;-\;{\vec{f}}_{0}||}_{\ell_{2}}^{2}$$

Then we have 

(18)

Some other preset parameters are

$$L\;=\;\frac{{||D||}^{2}}{\mu \sigma_{d}},\sigma_{d}\;=\;1$$

$$||\nabla f\left[i,j\right]||\;=\;{\left[{\left(\left(D_{h}f\right)\left[i,j\right]\right)}^{2}\;+\;{\left(\left(D_{v}f\right)\left[i,j\right]\right)}^{2}\right]}^{\frac{1}{2}}$$

### ART + MDATV scheme

In this paper, the data fidelity constraint is processed by ART iteration, and the MDATV regularization is minimized by NESTA. To use the prior information of the edges in multiple directions, the original ART + TV scheme [11] is not suitable for MDATV regularization. In the ART + TV scheme, firstly, the overall projection data are used to update the estimated image through ART. Then the estimated image is used as the input of the minimization method. The ART and the minimization is operated alternately. Each pair of successive ART and minimization constitutes an iteration of the ART + TV scheme. The workflow of the ART + TV scheme is shown below:

In the above scheme, the stopping criterion is the relative error between the estimated image and the true phantom image being less than 1.0 × 10^–5^. The relative error is computed as

$$\epsilon \;=\;\frac{||{\vec{f}}_{r}\;-\;{\vec{f}}_{0}||}{||{\vec{f}}_{0}||}$$

where ${\vec{f}}_{r}$ is the estimation of the restored image, ${\vec{f}}_{0}$ is the original phantom image known as ground truth. || · || is the Frobenius norm.

However, the MDATV use the prior information of edges in multiple directions by adjusting the angle θ  in (7). In the ART + TV scheme, there is no time to adjust θ . Therefore, the ART + MDATV scheme is proposed.

In the ART + MDATV scheme, projection data are divided into *m* groups based on the directions of the projection rays. In each group of projections, the projection rays are in the same direction for the parallel projection, and in the approximately same direction for the fan-beam projection. Thus each group of projections corresponds to an angle *θ*_*k*_ (*k* = 1, 2, …, *m*), which specifies the location of the X-ray source. Then for the *k*th group of projections, the ART and the minimization of MDATV regularization of *θ*_*k*_ are operated once successively. The estimated image of the *k*th group of projections is used as the initial estimate of the (*k* + 1)th group of projections. The process of the *m* groups of projections constitutes an iteration of the ART + MDATV scheme. The workflow of the ART + MDATV scheme is shown below:

The stopping criterion in the above scheme is the same as that in the ART + TV scheme. Based on the experiments, the iteration times of NESTA for MDATV is set as 10, and the iteration times of NESTA for TV and ATV are both set as 40.

## Simulations and experiments

Numerical simulations and real data experiments are conducted to validate the performance of MDATV based approaches. The TV and ATV based approaches are used for comparison. To compare the stableness of GD and NESTA, for each method, they are respectively used for minimizing the regularizations. Therefore, the combination of ART with three different regularizations and two kinds of minimization methods leads to six approaches: ART-TV-GD, ART-TV-NESTA, ART-ATV-GD, ART-ATV-NESTA, ART-MDATV-GD, and ART-MDATV-NESTA. The simulations for noisy measurements are conducted for comparing the noise robustness of different regularizations. Finally, the real data experiment is used to testify the effectiveness of MDATV in practical applications. Besides, this paper uses the CT module in “Image reconstruction toolbox” [38] to construct the projection matrix and operate the forward and backward projections.

### Numerical simulation

#### ***Simulation settings***

**Figure 5:**
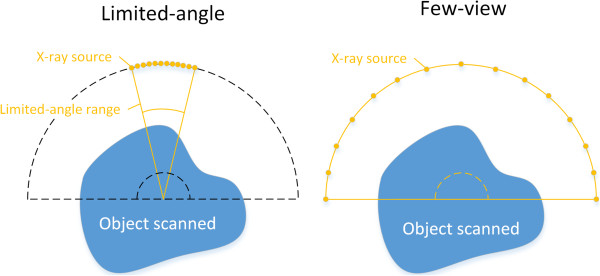
Schematic diagram of ‘limited-angle’ and ‘few-view’ sparse projection styles.

To reduce the X-ray dose, reduction of projection rays is a natural option. There are two common styles for reducing the projection rays. They are limited-angle [9] and few-view [10] styles. The schematic diagrams of these two styles are shown in Figure 5. For the same number of projection views, the information of target image in the few-view style is much more than that in the limited-angle style. In this paper, we only describes the simulations of the few-view style.The phantom used in this paper is generated by the function ‘phantom(128)’ in MATLAB^®^, as shown in Figure 3(a). It has 128 × 128 pixels. The simulated X-ray detector has 240 bins for the fan-beam projection. And the size of the detector bin is 2 millimeters. In the fan-beam projection, the distance between the source and center of the detector is 960.45 millimeters, and the distance from the source to the origin is 628.88 millimeters.

According to the ERP (Exact Reconstruction Principle) [6], if the number of FT (Fourier Transform) samples is twice the number of non-zero pixels in the gradient image, then the optimization program (1) can yield a unique solution. The gradient image of Shepp-Logan phantom is shown in Figure 3(d), where the number of non-zero pixel is 1743. In the digital condition, according to the central slice theory [34], one projection data corresponds to one FT sample. Therefore, we need at least 1743 × 2 = 3486 projection data points. While the fan-beam detector has 240 bins, thus based on ERP we need 3486 ÷ 240 ≈ 15 views of projections. For comparison, we also take some less views of projections, such as 11 and 13 views, in the numerical simulations.

The best step sizes of GD are different when the projection style or the regularization changes. Table 1 lists the best step sizes of GD for the fan-beam projections with different regularizations and projection views. These best step sizes are obtained by the traversal method within the accuracy of 1. Since the perturbation within 1 rarely affects the convergence rate of GD.

**Table 1:** The best step sizes of GD used in the numerical simulations

	**11 views**	**13 views**	**15 views**
TV	44	47	51
ATV	18	20	25
MDATV	4	4	4

To test the robustness of these methods to noise, the Poisson noise is introduced into the projections by the function ‘poisson’ in the “Image reconstruction toolbox” [38]. The incident photon number is set as 1.0 × 10^5^. Table 2 lists the best step sizes of GD for the noisy reconstructions.

**Table 2:** The best step sizes of GD used in the numerical simulations with noise

	**11 views**	**13 views**	**15 views**
TV	44	44	46
ATV	17	20	24
MDATV	4	4	3

#### ***Visualization-based evaluation***

**Figure 6:**
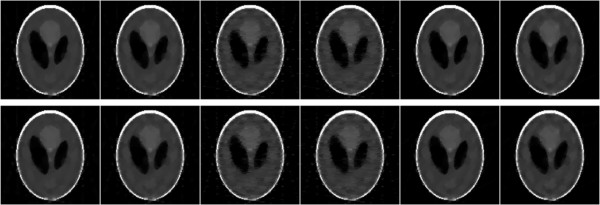
**Restored images from 11 views of noise-free (top row) and noisy projections (bottom row) with methods of ART-TV-GD, ART-TV-NESTA, ART-ATV-GD, ART-ATV-NESTA, ART-MDATV-GD, and ART-MDATV-NESTA (from left to right) after 100 iterations.** The display gray scale window is [0,1].

**Figure 7:**
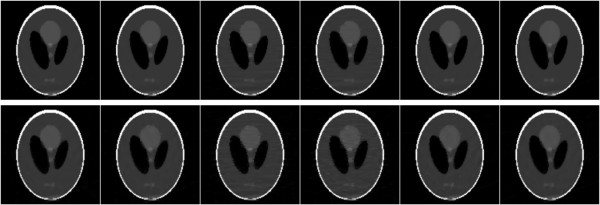
**Restored images from 15 views of noise-free (top row) and noisy projections (bottom row) with methods of ART-TV-GD, ART-TV-NESTA, ART-ATV-GD, ART-ATV-NESTA, ART-MDATV-GD, and ART-MDATV-NESTA (from left to right) after 100 iterations.** The display gray scale window is [0,1].

The restored images from 11 views of noise-free and noisy projections are shown in Figure 6, and the restored images from 15 views of noise-free and noisy projections are shown in Figure 7. Both the observations of Figures 6 and 7 indicate that the TV and MDATV regularizations are obviously better than ATV regularization. There are some horizontal artifacts in the restored image of ATV methods, which is caused by the unbalanced regularization, while the projection data are uniformly distributed. It can also be observed that increased projection data prominently improved the image quality.

#### ***Profile-based evaluation***

**Figure 8:**
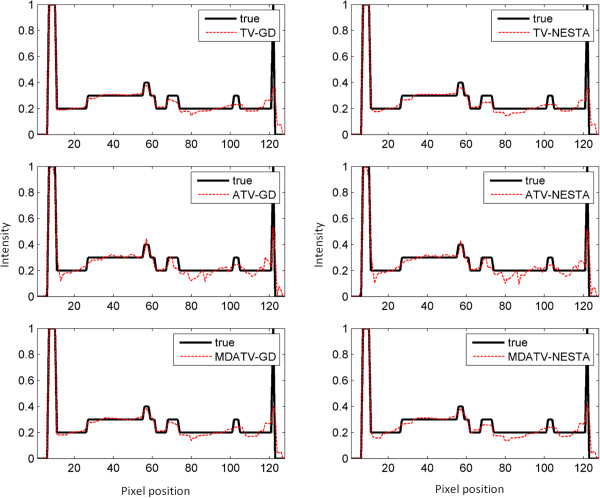
Vertical profiles (64th column, 1st row to 128th row) of the images restored from 11 views of noise-free projections.

**Figure 9:**
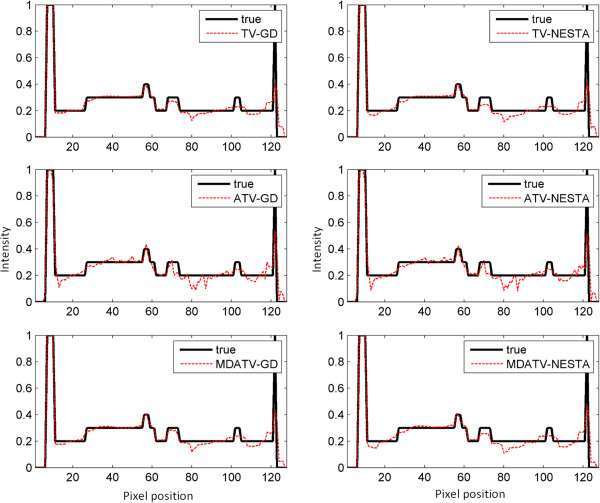
Vertical profiles (64th column, 1st row to 128th row) of the images restored from 11 views of noisy projections.

**Figure 10:**
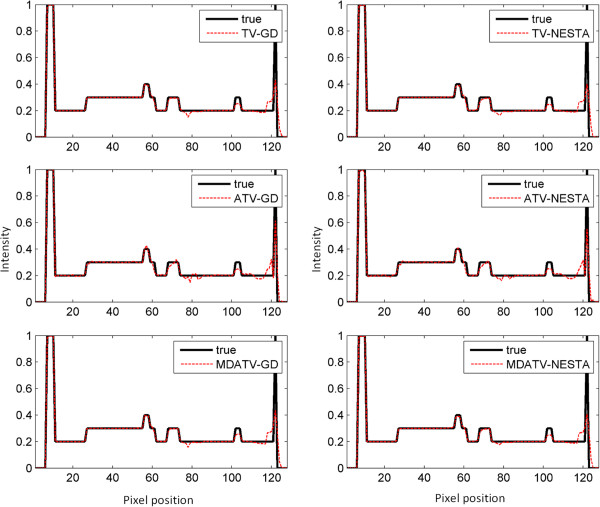
Vertical profiles (64th column, 1st row to 128th row) of the images restored from 15 views of noise-free projections.

**Figure 11:**
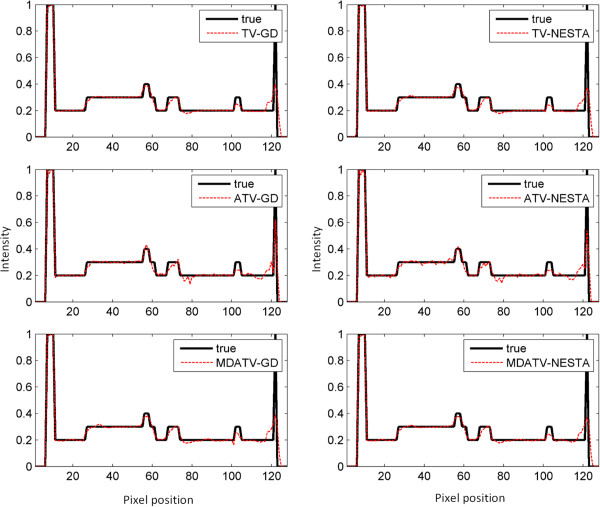
Vertical profiles (64th column, 1st row to 128th row) of the images restored from 15 views of noisy projections.

To further visualize the difference among various methods, vertical profiles of restored images were drawn across the 64th column, from the 1st row to the 128th row. Figures 8 and 9 shows the vertical profiles of the restored images in Figure 6 and the corresponding profile in the original Shepp-Logan phantom. And the vertical profiles of the restored images in Figure 7 and their corresponding profile in the original Shepp-Logan phantom are shown in Figures 10 and 11. The observations from all the profiles also indicate the same conclusion as the visualization-based evaluation.

#### Relative error study

**Figure 12:**
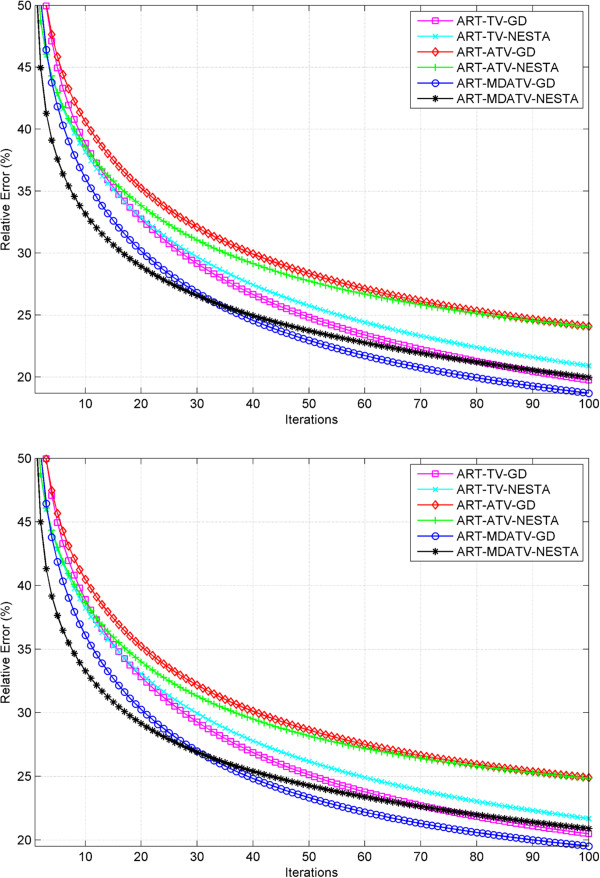
Relative error of reconstructions from 11 views of noise-free (top) and noisy (bottom) projections with six methods versus iterations.

**Figure 13:**
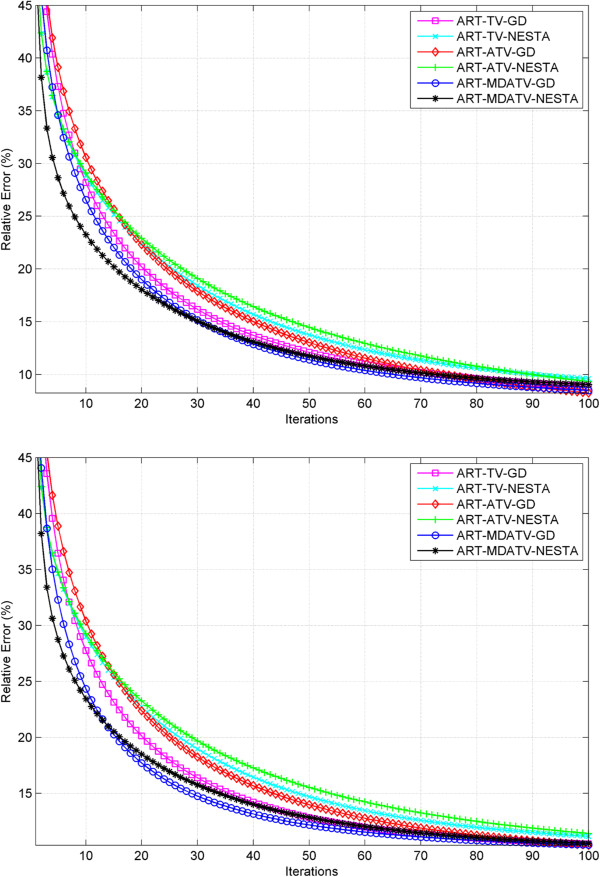
Relative error of reconstructions from 15 views of noise-free (top) and noisy (bottom) projections with six methods versus iterations.

The relative error is used in the simulation iterations as stopping criterion. Therefore, it can also be used to show the convergence condition of various methods. The curves of relative errors versus iteration times of reconstruction processes from 11 and 15 views of projections are shown in Figures 12 and 13. The observations indicate that after some iterations the reconstructions would converge to steady conditions. And the relative error after the final iteration can represent the reconstruction quality. For the 11 views condition, MDATV is better than TV, and TV is better than ATV. While for the 15 views condition, the three regularizations are hard to distinguish.

#### UQI (Universal Quality Index) study

**Figure 14:**
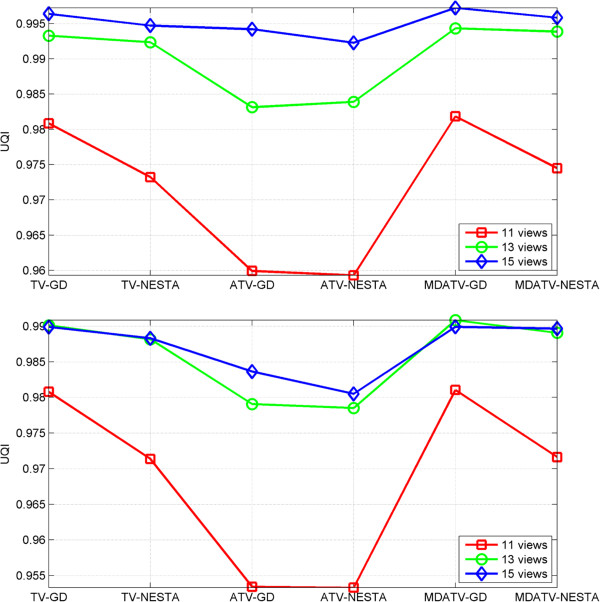
UQI values of restored images by various methods from 11, 13 and 15 views of noise-free (top) and noisy (bottom) projections.

To perform more quantitative analysis of these methods, the UQI [39] is introduced. UQI measures the similarity between the desired image and its ground truth image [23]. UQI value ranges between zero and one. A higher UQI value represents a higher similarity between the testing image and the ground truth image, and vice versa. The ROI (Region Of Interest) used for computing UQI is in the red rectangle as shown in Figure 3(a). The UQI values of restored images by various methods from different views of projections are plotted as several curves shown in Figure 14. The UQI values are in accord with the relative errors. The observations also indicate that the MDATV is better than TV, and TV is better than ATV. When the volume of projection data increase, the distinctions among these regularizations are decreasing.

### Real data experiment

**Figure 15:**
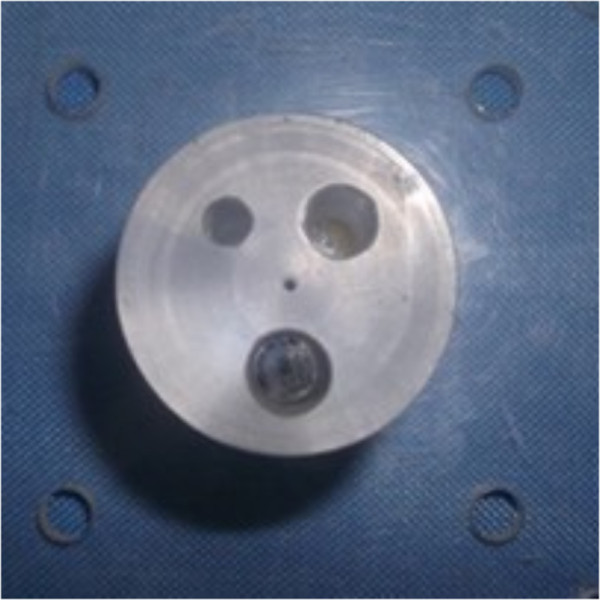
Cross section of the organic glass column with three holes in real data experiment.

To demonstrate the effectiveness of proposed method in real CT application, the real data experiment is conducted. The projection data is from a micro-CT machine in Tianjin Key Laboratory of Biomedical Detecting Techniques and Instruments. The scanning phantom is an organic glass column with three holes of different diameters, as shown in Figure 15. In this experiment, the left hole on top is empty (filled with air), the right hole on top is filled with corn flour, and the bottom hole is filled with a copper bar. The distance between the source and center of the detector is 960.45 millimeters, and the distance from the source to the origin is 628.88 millimeters.

**Figure 16:**
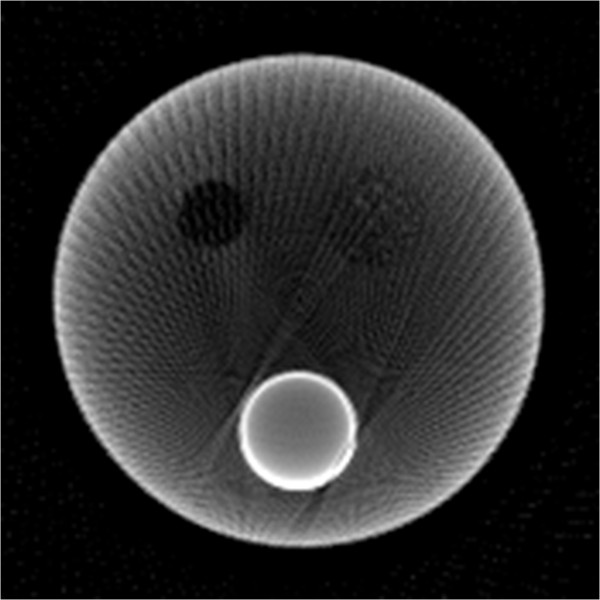
**Restored iamges from 120 views of fan-beam projections with FBP.** The display gray scale window is [80,400].

In the experiment, the detector has 1024 bins, and the physical size of each bin is 0.05 millimeters. Therefore, the raw sinogram of one view has 1024 pixels, and each pixel represents the physical length of 0.05 millimeters. However, the computation load of the iterative reconstruction is very heavy for the high resolution sinogram. Thus the high resolution sinogram is down sampled by the ratio of 4. And the low resolution sinogram of one view has 256 = 1024 ÷ 4 pixels. To maintain the physical length of the projection geometry, each pixel in the low resolution sinogram represents the physical length of 0.2 = 0.05 × 4 millimetres. Actually, in this down sampling, four neighboring pixels in the raw sinogram of one view is averaged as one pixel in the low resolution sinogram of one view.In this experiment, restrained by the micro-CT machine, the whole projection data contains 120 views of fan-beam projection uniformly distributed across 360°. Its FBP reconstruction is shown in Figure 16. Compare Figure 15 and 16, it is easy to distinguish the air and copper filled holes from the background organic glass, while the corn filled hole is hard to distinguish from the background organic glass. This is because that the attenuation coefficient of corn flour is very similar to that of the organic glass, while the attenuation coefficients of air and copper are very different from that of the organic glass.

**Figure 17:**
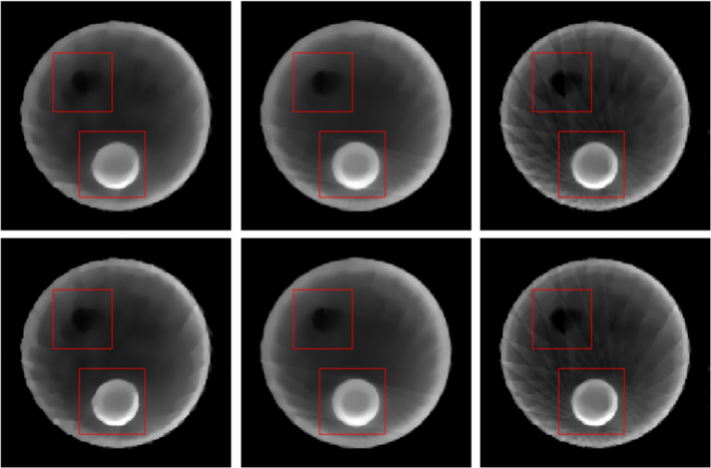
**Restored images from 30 views of few-view style fan-beam projections after 5 iterations (top row) and 100 iterations (bottom row) with three methods (from left to right): ART-TV-GD, ART-MDATV-GD, ART-MDATV-NESTA.** The display gray scale window is [80,400].

**Figure 18:**
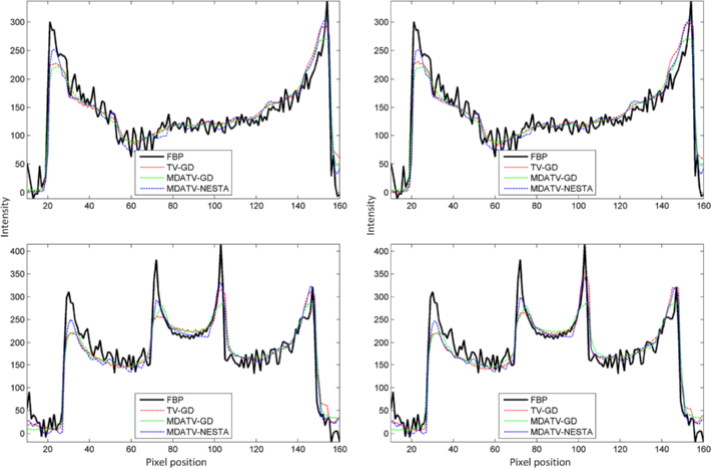
Horizontal profiles (63rd row (top) and 127th row (bottom), 1st column to 128th column) of the restored images after 5 (left) and 100 (right) iterations.

**Figure 19:**
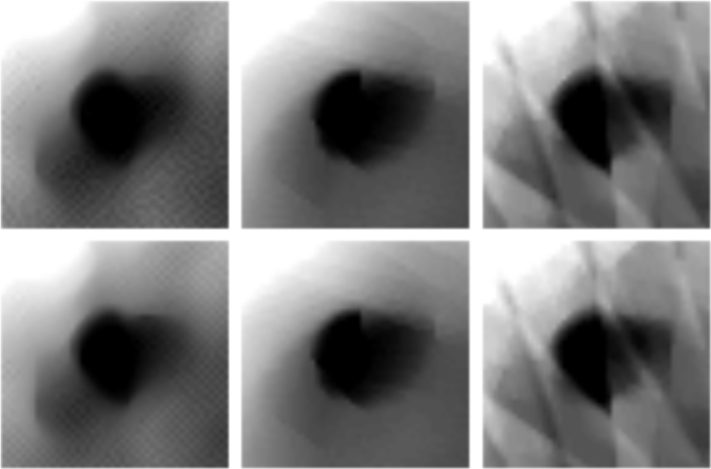
**Restored circles representing the air filled hole in Figure**17**.**

**Figure 20:**
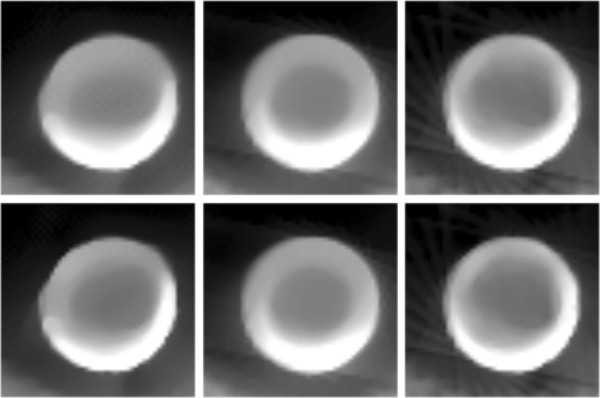
**Restored circles representing the copper filled hole in Figure**17**.**

For the sparse CT reconstruction experiment, 30 views of fan-beam projections are chose uniformly across the range of 360°. Due to the ATV’s poor performance for the sub-ERP projections (volume of projections is less than that required by ERP), we only compare TV and MDATV based methods. The reconstruction methods used in the experiments are ART-TV-GD, ART-MDATV-GD, and ART-MDATV-NESTA. The step sizes of GD used in the reconstructions are 92 for TV and 10 for MDATV, which are estimated from an approximate synthetic phantom. The iteration times of NESTA for MDATV is 40.In Figure 17, we show the reconstructions after 5 iterations (top row) and 100 iterations (bottom row) with three methods (from left to right): ART-TV-GD, ART-MDATV-GD, ART-MDATV-NESTA. In the restored images, the holes filled with air and copper are distinct, while the hole filled with corn is undistinguishable. To further visualize the difference among various methods, horizontal profiles of restored images were drawn across the 63rd row (for the air and corn filled holes) and 127th row (for the copper filled hole), from the 10th column to the 160th column. The profiles of restored images after 5 and 100 iteration are shown in Figure 18. Since the projection data is not sufficient, the FBP reconstructions abound with artifacts, but the FBP reconstructions clearly depict the boundaries of the air and copper filled holes. And the observations from Figure 18 indicate that MDATV-NESTA profiles matches the FBP profiles best.The regions of holes filled with air and copper (in the red rectangles in Figure 17) are zoomed in and shown in Figures 19 and 20 respectively. To increase the contrast of Figures 19 and 20, 5% of data is saturated at low and high intensities of the original image. Figures 19 and 20 indicate that ART-MDATV-GD method gave the best results. Since the restored circles by ART-MDATV-GD are the most round. The restored circles of ART-MDATV-NESTA are more round than that of ART-TV-GD. The radial artifacts in the restored images are metal artifacts, which can be removed by some off-the-shelf methods.

## Discussions and conclusions

The simulations and experiments both indicate that MDATV is a useful and robust regularization for the sparse CT reconstruction when the volume of the projection data is less than the volume required by the ERP. But actually, it is impossible to satisfy the ERP in practical applications. Because the practical target images are not piecewise-constant, which means the non-zero elements of the gradient image are infinite. Thus the volume of digital projections is always not enough. Therefore, using MDATV to incorporate more a prior information of the target images is valuable and gainful in practical applications, as shown in the real data experiment. And the indistinguishable results among the three regularizations with ERP projections in the simulations is because that the synthetic phantom is piecewise-constant, which means its ERP samples are limited. Obviously, when the projections’ volume satisfies the ERP, there is no need to incorporate any other prior information.

Since MDATV can represent edges in any direction, the MDATV based methods can use the prior information of edge directions more efficiently than TV or ATV based methods. Therefore, MDATV based methods could enhance the edges in the projection rays directions and suppress the potential artifacts in the no projection rays directions, which leads to the improvement of the reconstructions.

In this paper, NESTA is proposed to minimize the MDATV regularization instead of GD. This is because that NESTA gives almost the same results as GD does for minimization of the regularizations, but NESTA is more stable than GD. For example, Tables 1 and 2 list the best step sizes of GD used in the simulations. The best step size of GD changes much for different projections and noisy conditions. And there is no rule to obtain the best step size of GD in advance. Nevertheless, the few parameters in NESTA almost does not change in the simulations and experiments. Obviously, NESTA is a considerable choice for minimizing the regularizations in the sparse CT reconstruction.

The accumulated computation time of the simulations are listed in Table 3. It is observed that the computation load of NESTA is a little higher than that of GD, but NESTA is more stable than GD. If the comparison includes the time of finding the best step size of GD, then NESTA will be much faster than GD. Due to the special scheme of ART + MDATV, MDATV related methods need more computation time than TV or ATV related methods. This is because that MDATV related methods do the minimization after each group of ART iteration. If the projections are classified into *m* groups, then MDATV related methods have *m* - 1 times of minimization more than TV or ATV related methods. Furthermore, each minimization has several times of GD or NESTA iterations.

**Table 3:** Accumulated computation time of numerical simulations

	**TV-GD**	**TV-NESTA**	**ATV-GD**	**ATV-NESTA**	**MDATV-GD**	**MDATV-NESTA**
11 views	43.15	59.20	45.44	58.75	84.41	95.30
13 views	51.44	69.29	53.27	68.88	99.53	118.68
15 views	58.26	74.75	59.57	75.37	114.43	137.35
11 views noisy	45.67	59.83	47.02	60.04	84.57	96.14
13 views noisy	51.49	67.90	52.84	68.13	100.07	115.10
15 views noisy	58.79	76.39	60.26	74.62	114.24	131.08

(unit: second).

In addition, it is notable that the fan-beam geometry in the numerical simulations and real data experiment are the same, both from the geometry of the micro-CT machine. In this geometry, the view angle of the fan-beam projection is so small that the projection rays in the same projection view are approximately in the same direction. While in practical applications, the view angle of the fan-beam projection may be bigger so that the projection rays in the same projection view are not approximately in the same direction. In this condition, it needs to rearrange the projection data according to their corresponding projection directions, such that the projection rays having the approximately same direction can be classified into the same group. This will be one of our future research directions.

This paper proposed the MDATV regularization to sufficiently use a prior information of projection directions and image sparsity in the sparse CT reconstruction. Due to the effective use of prior information of projection directions, the restored edge information is greatly enhanced, and thus to improve the reconstructions. The numerical simulations and real data experiments demonstrate the advantage of MDATV over other regularizations. NESTA is proposed as an alternative to GD for minimizing the TV-based regularizations. Because NESTA is more stable and has the almost same performances as GD.

Otherwise, although the MDATV regularization method was only validated for fan-beam projections, it is simple to introduce this regularization approach to other tomography reconstructions. And using prior information of projection directions may further facilitate the development of tomography. Besides, we think the presentation of edges in multiple directions may have more applications in the fields of image enhancement and reconstruction.

## Abbreviations

MDATV: Multi-direction anisotropic total variation; CT: Computed tomography; ATV: Anisotropic total variation; TV: Total variation; 2D-IGS: 2-Dimensional image gradient space; ART: Algebraic reconstruction technique; NESTA: NESTerov’s algorithm; GD: Gradient descent; IRIS: Iterative reconstruction in image space; ASiR: Adaptive statistical iterative reconstruction; GE: General electric Co; iDose: trademark of Philips; AIDR: Adaptive iterative dose reduction; FBP: Filtered back projection; ART-TV-MIN: Algebraic reconstruction technique total variation MINimization; ASD-POCS: Adaptive steepest descent projection onto convex sets; PICCS: Prior image constrained compressed sensing; PIRPLE: Prior image registered penalized likelihood estimation; FCCS: Feature constrained compressed sensing; TpV: Total p-variation; GPU: Graphics processing unit; CG: Conjugate gradient; GPBB: Gradient projection Barzilai Borwein; ADMM: Alternating direction method of multipliers; POCS: Projection onto convex sets; ERP: Exact reconstruction principle; FT: Fourier transform; UQI: Universal quality index; ROI: Region of interest.

## Competing interests

The authors declare that they have no competing interests.

## Authors’ contributions

HL and XC conceived the study. HL implemented the studies and drafted the manuscript. XC, YW, and DY contributed to discussions and suggestions throughout this work. XC and DY supervised the study. ZZ and QZ designed and performed the experiments. All authors read and approved the final manuscript.
